# Simple multi-wavelength imaging of birefringence:case study of silk

**DOI:** 10.1038/s41598-018-36114-8

**Published:** 2018-12-05

**Authors:** Reo Honda, Meguya Ryu, Jing-Liang Li, Vygantas Mizeikis, Saulius Juodkazis, Junko Morikawa

**Affiliations:** 1 Tokyo Institute of Technology, Meguro-ku, Tokyo 152-8550 Japan; 2 Institute for Frontier Materials, Deakin University, Geelong, Victoria 3220 Australia; 3 Research Institute of Electronics, Shizuoka University, Naka-ku, 3-5-3-1 Johoku, Hamamatsu, Shizuoka 4328561 Japan; 4 Swinburne University of Technology, John st., Hawthorn, 3122 Vic Australia; 5 Melbourne Center for Nanofabrication, Australian National Fabrication Facility, Clayton, 3168 Melbourne Australia

## Abstract

Polarised light imaging microscopy, with the addition of a liquid crystal (LC) phase retarder, was used to determine the birefringence of silk fibres with high (∼1 *μ*m) spatial resolution. The measurement was carried out with the silk fibres (the optical slow axis) and the slow axis of the LC-retarder set at parallel angles. The direct fit of the transmission data allowed for high fidelity determination of the birefringence *Δn* ≈ 1.63 × 10^−2^ (with ∼2% uncertainty) of the brown silk fibre, (*Antheraea pernyi*) averaged over the wavelength range *λ* = (425–625) nm. By measuring retardance at four separate wavelengths, it was possible to determine the true value of the birefringence of a thicker sample when an optical path may include a large number of wavelengths. The numerical procedures and required hardware are described for the do-it-yourself assembly of the imaging polariscope at a fractional budget compared to commercial units.

## Introduction

Optical imaging of metasurfaces for defining the engineered birefringence and its orientational pattern is gaining rapid interest due to the ability to directly evaluate the fabrication quality and phase retardance of flat optical elements using transmission polariscopy^1^. This method was previously demonstrated to be able to determine the slow axis orientation and retardance of any arbitrary sample^2^ and was commercially implemented as a side-port addition onto a microscope (Abrio). A wider use of this technique, however, was hampered by its comparatively high price and production of the unit was consequently discontinued. Nevertheless, there is a need for the *in situ* monitoring of birefringence in complex micro-fluidic flow cells^3^ and cell division microscopy where optical detection of cell division can be monitored in real time^4^ using such simple instrumentation. Measurements of birefringence are highly necessary in microscopy and material science fields, however, expensive dedicated microscopes or bulky add-on microscopy units must be used which generally only operate at fixed wavelengths^2^. Birefringence can be inferred from Stokes polarimetry which is realised by different principles of phase delay or polarisation rotation *e.g*. based on photoelasticity^5^ or the use of LC retarders^6,7^. When waveplates are used together with rotating elements and lock-in amplifiers, setups of high sensitivity and resolution can be created. A crossed polariser-analyser setup with a rotating quarter-waveplate compensator was recently used for determination of 3D orientation maps of birefringent fibre structures in brain tissue^8^. These custom set-ups, however, can become bulky, complex and, frequently, wavelength specific.

**Figure 1:**
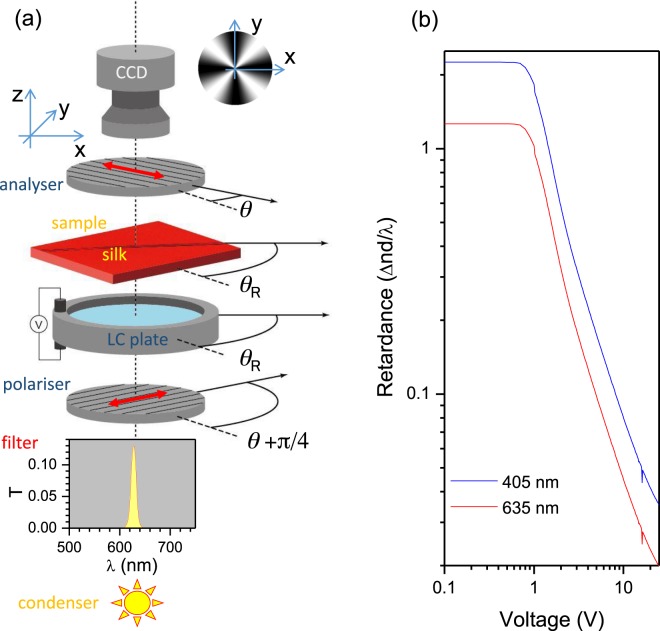
(**a**) Schematic presentation of the assembled setup. The Maltese cross (shown near CCD) is a polar plot of the mean intensity with variable *θ* − *θ*_*R*_ (Eqn. 1). A 10-nm-bandwidth filter (see the actual spectral profile of transmission *T* plotted) was inserted to filter out the rest of the white light condenser illumination. Arrows mark transmission orientation of the polariser and analyser. (**b**) Retardance *vs*. voltage of LC-cell (LCC1223T-A; Thorlabs) at two wavelengths calibrated by the manufacturer.

Simplification of the optical retardance measurement over a broader spectral range remains a requirement for flat optical elements and bio-materials which display high orientational anisotropy and domain structure. For example, the birefringence of silk is usually measured by shear interferometry^9–11^, which is not capable of high resolution imaging. The emerging optical applications which utilise transparent wood^12^ also require better understanding of the optical properties of the micro-tubular wood structure. Stress-induced birefringence in crystals/glasses/polymers^13–15^, volume phase transitions^16^, and complex topological structures for volumetric stress control^17^, or patterning of absorbance in transparent materials^18^ all produce complex optical anisotropy which demands high resolution ∼*λ* imaging. A set-up consisting of a crossed polariser and analyser can be used to reveal the birefringence Δ*n* of a sample placed between them using optical imaging at a selected wavelength, *λ*. The retardance Δ*nd* is defined by the thickness of the sample *d* and birefringence Δ*n*. The transmittance *T* through the birefringent medium of thickness *d*, when reflectance and absorbance are negligible for the crossed polariser and analyser, is given by (Fig. 1(a)):

1 $${T}_{\theta }={I}_{\theta }/{I}_{0}={\sin }^{2}\mathrm{2(}\theta -{\theta }_{R}){\sin }^{2}(\pi {\rm{\Delta }}nd/\lambda ),$$

where *I*_*θ*_ and *I*_0_ are the transmitted and incident intensities, respectively, *θ* is the angle between the transmission axis of the analyser and the horizontal x-direction of the field of view where it is also positive for the anti-clockwise rotation (looking into the beam), *θ*_*R*_ is the slow (or fast) axis direction with the slow axis^19^ usually aligned to the main molecular chain or along the polymer stretch or, in this study, aligned along the length of the silk fibre. Equation 1 represents the Maltese cross pattern shown in Fig. 1(a).

In this study, we used a simple LC-cell (Fig. 1(a)), which has an electrically controlled birefringence (Fig. 1(b)), as a compensator for the determination of the birefringence of a single silk fibre. The silk sample was placed directly on the LC-cell window and aligned with the slow axis of the LC-retarder. Transmission vanished at the regions with zero birefringence (Eqn. 1). Data acquisition and analyses were fully automated using Labview and Matlab scripts. By using standard microscopy imaging at a freely chosen wavelength, the birefringence of a single strand of silk was determined with high fidelity, achieving a resolution which is comparable to the wavelength *λ* at tight focusing. Therefore, this method can potentially be used to measure the birefringence of any transparent material over the visible 400–800 nm spectral range which is determined by the transparency of the LC-cell. Due to the virtue of multi-wavelength measurement capability, the method used in this study allows to determine the birefringence of a material even when the retardance possesses wavelength changes.

## Method and Samples

### Polarisation change due to birefringence

The setup used in this study was based on illumination of the sample using linearly polarised light (Fig. 1(a)). Simple Jones matrix calculus^20,21^ (as compared to the more general Mueller calculus) was applied to determine the evolution of the electric field of light as outlined next. The x-polarised (horizontally) incident light can be defined by the E-field Jones vector (Fig. 1):

2 $${E}_{H}=(\begin{array}{c}1\\ 0\end{array}).$$

The analyser is crossed and can only transmit y-polarised (vertical) light. The corresponding Jones matrix is given by:

3 $${J}_{V}=(\begin{array}{cc}0 & 0\\ 0 & 1\end{array}).$$

A generic Jones matrix of the retarder with the phase delay *ϕ* = *k*Δ*nd*, wavevector *k* = 2*π*/*λ*, and with the slow axis at angle *θ* with respect to the x-axis is given by:

4 $${J}_{R}(\varphi ,\theta )=(\begin{array}{cc}\cos \,\frac{\varphi }{2}+i\,\sin \,\frac{\varphi }{2}\,\cos \,\mathrm{(2}\theta ) & i\,\sin \,\frac{\varphi }{2}\,\sin \,\mathrm{(2}\theta )\\ i\,\sin \,\frac{\varphi }{2}\,\sin \,\mathrm{(2}\theta ) & \cos \,\frac{\varphi }{2}-i\,\sin \,\frac{\varphi }{2}\,\cos \,\mathrm{(2}\theta )\end{array}).$$

During the measurements, the LC-retarder was inserted with the silk fibre oriented parallel to the slow axis of the LC-retarder (Fig. 1). Eqn. 4 was used for the LC-retarder *J*_*LC*_(*ϕ*, *θ*). The setup and calibration curves for the retardance *vs*. voltage is shown in Fig. 1. The silk fibre contribution to the phase retardance is given as *J*_*s*_(*ϕ*_*s*_, *θ*_*s*_), where *θ*_*s*_ is calculated from the x-axis (*θ*_*s*_ = 0). The overall transmission through the setup (Fig. 1(a)) can therefore be determined by the following equation:

5 $${E}_{t}=({J}_{V}{J}_{s}{J}_{LC}){E}_{H}=(\begin{array}{cc}0 & 0\\ A & B\end{array})\,{E}_{H}=(\begin{array}{c}0\\ A\end{array}),$$

The analytical expressions for the coefficients of *A* = *a*_1_ + *ia*_2_ and *B* = *b*_1_ + *ib*_2_ are given by:

$$\begin{array}{rcl}{a}_{1} & = & \sin \,\frac{{\varphi }_{s}}{2}\,\sin \,\frac{{\varphi }_{LC}}{2}(\cos \,\mathrm{(2}{\theta }_{s})\sin \,\mathrm{(2}{\theta }_{LC})-\,\sin \,\mathrm{(2}{\theta }_{s})\cos \,\mathrm{(2}{\theta }_{LC}))\\ {a}_{2} & = & \sin \,\frac{{\varphi }_{s}}{2}\,\cos \,\frac{{\varphi }_{LC}}{2}\,\sin \,\mathrm{(2}{\theta }_{s})+\,\cos \,\frac{{\varphi }_{s}}{2}\,\sin \,\frac{{\varphi }_{LC}}{2}\,\sin \,\mathrm{(2}{\theta }_{LC})\\ {b}_{1} & = & -\,\sin \,\frac{{\varphi }_{s}}{2}\,\sin \,\frac{{\varphi }_{LC}}{2}\,\sin \,\mathrm{(2}{\theta }_{s})\sin \,\mathrm{(2}{\theta }_{LC})+\,\cos \,\frac{{\varphi }_{s}}{2}cos\frac{{\varphi }_{LC}}{2}\\  &  & -\,sin\frac{{\varphi }_{s}}{2}\,\sin \,\frac{{\varphi }_{LC}}{2}\,\cos \,\mathrm{(2}{\theta }_{s})\cos \,\mathrm{(2}{\theta }_{LC})\\ {b}_{2} & = & -\,\cos \,\frac{{\varphi }_{s}}{2}\,\sin \,\frac{{\varphi }_{LC}}{2}\,\cos \,\mathrm{(2}{\theta }_{LC})-\,\sin \,\frac{{\varphi }_{s}}{2}cos\frac{{\varphi }_{LC}}{2}\,\cos \,\mathrm{(2}{\theta }_{s}).\end{array}$$

The intensity of the transmission image detected by the CCD camera (Fig. 1(a)) is thereby given as *I* = AA^*^, where A^*^ is the complex conjugate. Further simplification of the trigonometric expressions *A*,*B* occurs at *θ*_*LC*_ = *θ*_*s*_ = *π*/4 and allows for the simple calculation of the intensity at each [*x*, *y*] pixel *I*(*x*, *y*). By matching *I*(*x*, *y*) with the experimentally measured retardance $Re{t}_{exp}\equiv \arcsin \sqrt{{T}_{\theta }}\mathrm{/2}\pi$ (Eqn. 1), the retardance Δ*n* × *d* can be determined. Furthermore, when the thickness of the birefringent region *d* is known (measured independently), the birefringence Δ*n* at each pixel can be calculated.

### Samples and measurements

A Nikon Optophot-POL microscope with an Olympus LMP PlanFLN objective lens with 20× magnification and numerical aperture *NA* = 0.4 was used for all measurements in this study. The optical images with VGA resolution 480 × 640 pixels were captured with a CCD camera BU030C Toshiba teli for analyses at *N* = 718 number of points (voltage values of the LC-retarder cell). The LC-retarder (LCC1223T-A, Thorlabs) was used in conjunction with the LCC25 controller and a temperature stabilizer TC200. The temperature stabilizer was used for the quantitative determination of the retardance. Factory calibration of retardance *vs*. applied voltage at selected wavelengths was provided by the vendor (Fig. 1(a)), however, we applied a different calibration procedure suitable for each wavelength selected by the bandpass filter. Image acquisition at different LC-retardance (number of points *N* = 718) was computer controlled and a Matlab code was used for the final image analysis using the protocol described in Sec. 2.1.

Two types of silk, white *Bombyx mori* and brown *Antheraea pernyi*, were used in this study. Prior to the experiment, both silk fibres were degummed, *i.e*. the sericin cladding was dissolved as described previously^22^ and single strands were used for imaging. Both types of silk have similar composition and structure, therefore, they should possess similar birefringence^22^. Brown silk strands have, on average, a slightly larger diameter. Image acquisition was carried out at standard room temperature and pressure.

### Calibration of retardance of LC-cell

Factory calibration of the retardance *vs*. applied voltage was provided by the vendor, however, the LC-retarder is sensitive to temperature and wavelength. Therefore, precise calibration is required for each measurement. The light intensity at “Air” ROI was utilized for the calibration.

The retardance of LC-cell *Ret*_*LC*_ is the function of applied voltage *V*. When the slow axis of LC-retarder is set to *π*/4 radian between the polariser and analyser of the microscope, the light intensity *I*_*Air*_ at the “Air” ROI (out of sample) is given by:

6 $${I}_{air}=({I}_{Air}^{max}-{I}_{Air}^{min}){\sin }^{2}(\pi Re{t}_{LC}(V))+{I}_{Air}^{min},$$

where ${I}_{Air}^{max}$ and ${I}_{Air}^{min}$ are the maximum and minimum light intensity *I*_*air*_. Retardance change by the applied voltage *V* is continuous as shown in Fig. 1(b). Using Eqn. 6, the retardation can be obtained from the intensity by the following equation:

7 $$Re{t}_{LC}=\{\begin{array}{ll}\frac{1}{\pi }\arcsin \sqrt{\frac{{I}_{Air}-{I}_{Air}^{min}}{{I}_{Air}^{max}-{I}_{Air}^{min}}} & ({V}_{max}\le V)\\ 1-\frac{1}{\pi }\arcsin \sqrt{\frac{{I}_{Air}-{I}_{Air}^{min}}{{I}_{Air}^{max}-{I}_{Air}^{min}}} & ({V}_{min} < V < {V}_{max})\\ 1+\frac{1}{\pi }\arcsin \sqrt{\frac{{I}_{Air}-{I}_{Air}^{min}}{{I}_{Air}^{max}-{I}_{Air}^{min}}} & (V\le {V}_{min}),\end{array}$$

where *V*_*max*_ and *V*_*min*_ are the applied voltage when light intensity *I*_*Air*_ is equal to ${I}_{Air}^{max}$ or ${I}_{Air}^{min}$. We can calculate *Ret*_*LC*_ from *I*_*Air*_ by Eqn. 7. As a result, calibration of *Ret*_*LC*_ was carried out by measuring light intensity *I*_*Air*_ when applied voltage *V* changes using Eqn. 7.

## Results and Discussion

First, we show a *qualitative* method of retardance imaging using silk fibres. Then, a *quantitative* method is demonstrated using a simple LC-retarder cell without employing waveplates (Sec. 2.1).

### Qualitative imaging of retardance

**Figure 2:**
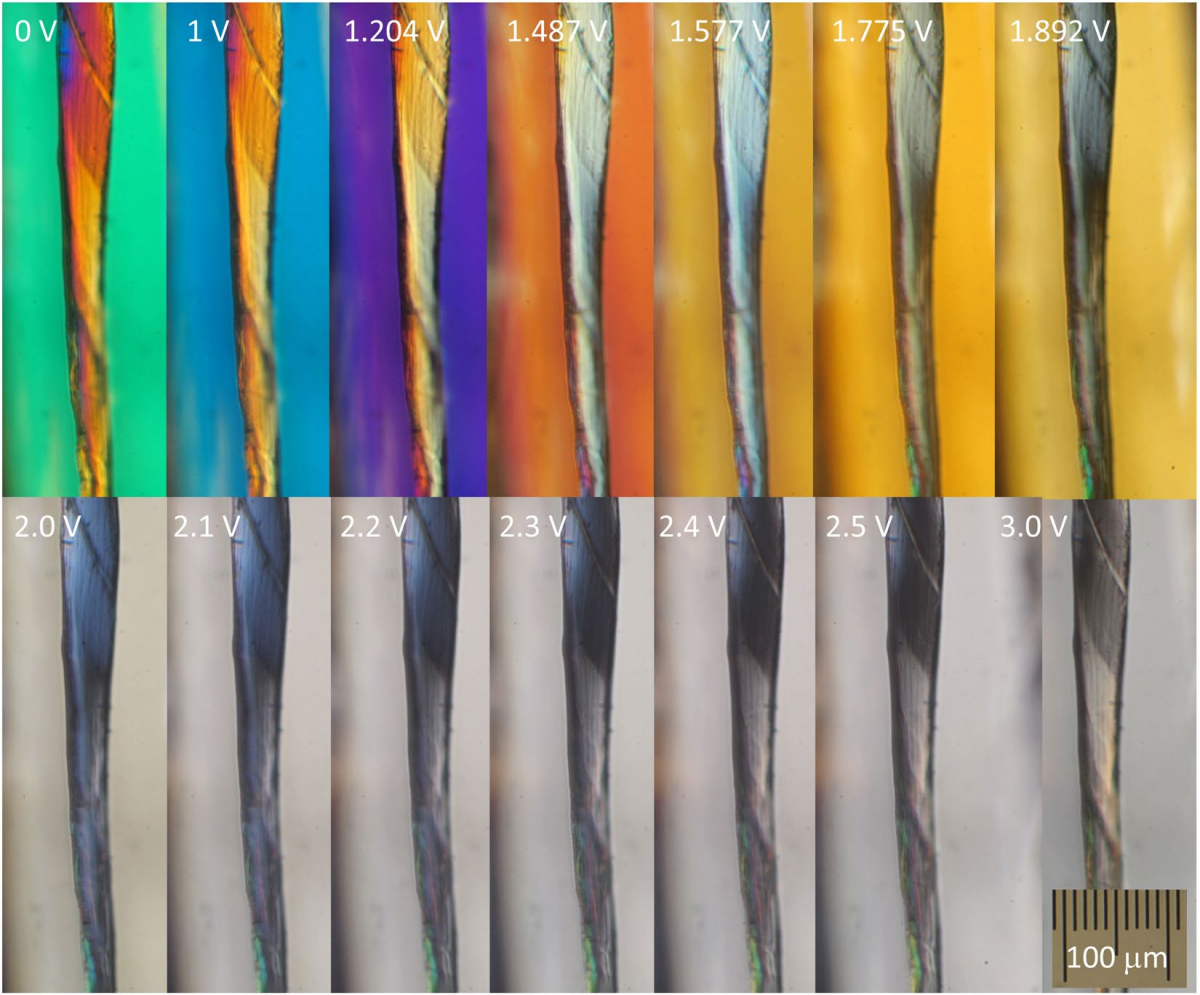
Optical images of white silk *Bombyx mori* captured through the crossed polariser-analyser set-up under white light illumination. The LC-retarder voltage is marked. The slow axis of the LC-retarder was set at *θ* = *π*/4 and the silk fibre was perpendicular to the slow axis of the LC-retarder.

Phase retardance is used in polarisation microscopy to create colour contrast under a white light (condenser) illumination. This is useful for the qualitative distinction of regions with different birefringence (phase thickness) in the image. Figure 2 shows images of a single white silk *Bombyx mori* fibre after degumming taken at different LC-retarder voltages. The slow axis of the LC-retarder was aligned perpendicularly to the silk fibre in order to counteract the birefringence by decreasing the retardance at larger voltages (Fig. 1(a)). When the birefringence Δ*n* was corrected the polar plot of the mean intensity formed a pattern similar to the Maltese cross (Fig. 1(a)). The darkest region in each image was observed to change when capturing images at larger voltages (Fig. 2). It is presumed that the triangular or trapezoidal shape of the cross section of the silk fibre was contributing to the non uniform colour appearance over the entirety of the fibre. Nevertheless, only a qualitative estimate of the birefringence can be made using this method, even when imaging is carried out at one wavelength or at a spectrally narrow bandwidth.

### Quantitative imaging of retardance

**Figure 3:**
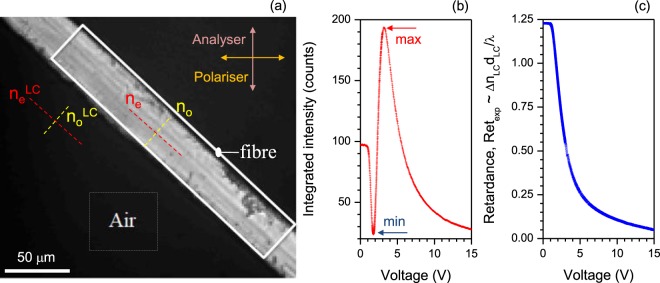
(**a**) Optical image of a brown silk *Antheraea pernyi* fibre captured while the LC-retarder cell voltage was set to 15 V. The bandpass filter at 625 nm wavelength was used for the white light condenser illumination. The birefringence of silk was determined by the difference between the extraordinary and ordinary refractive indices Δ*n* = *n*_*e*_ − *n*_*o*_. The dark region (the region outside of the fibre sample) is where the LC-retarder lies between the crossed polariser-analyser and is marked as the region of interest (ROI): “Air”. (**b**) The intensity integrated over selected “Air” (see, (**a**)) *vs*. the voltage (rms) of the retarder. (**c**) Digitalised retardance of the LC-cell $Re{t}_{exp}=\arcsin \sqrt{{T}_{\theta }}\mathrm{/2}\pi$, where *T*_*θ*_ = *I*_*θ*_/*I*_0_ (Eqn. 1). The temperature of the LC-retarder was set to 24.7–24.9 °C, the exposure time was 0.85 s.

**Figure 4:**
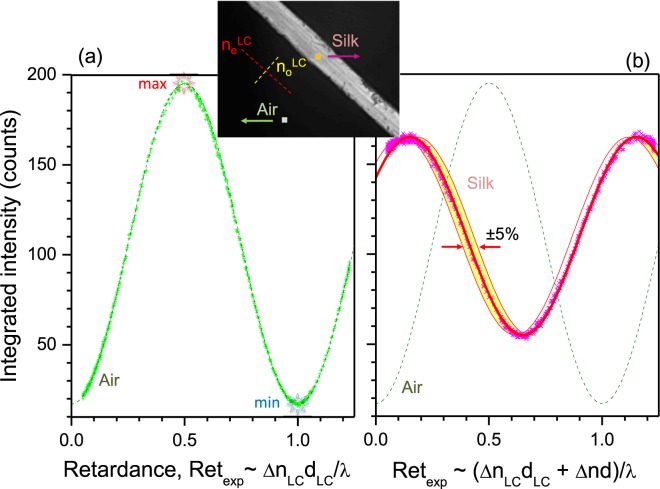
Experimental measurement of the transmission with 625 nm bandpass filter. (**a**) Integrated transmission intensity *vs*. retardance $Re{t}_{exp}=\arcsin \sqrt{{T}_{\theta }}\mathrm{/2}\pi$ for the marked region (see the inset) without silk fibre (marked square: “Air”). The inset image shows an optical micrograph of the brown silk *Antheraea pernyi* fibre at 15 V with highlighted regions of the birefringence measurement 2 × 2 pixels (0.66 × 0.66 *μ*m^2^). The resolution of the objective lens was ∼1 *μ*m at a wavelength of 625 nm. (**b**) Integrated transmission through the silk fibre (dots) and the best fit (line). The fit function $fit(x)=a{sin}^{2}(\pi [x+b])+o$, where the best fit was achieved for selected amplitude, phase delay, and offset *a* = 110, *b* = 0.35, and *o* = 55.

In this section, we describe the determination of the birefringence with good reproducibility, using CCD camera imaging at different LC-retarder voltages (number of points *N* = 718) at different *ϕ*_*LC*_ values and by application of the formulas in Eqns 2–5. We used a ∼10 nm bandwidth filter to select a narrow spectral window from the white light condenser illumination. The silk fibre was set at an angle of *π*/4 radian between the polariser and analyser (Fig. 3). Additionally, the fibre was set parallel to the slow axis of the LC-retarder (*θ* − *θ*_*R*_) = *π*/4. A region of interest (ROI) “Air” was selected outside the silk fibre (Fig. 3(a)) where only the reference retardance of the LC-cell was present in the optical path. The *N* = 718 number of measurement points of transmittance were selected in equidistant steps of retardance over the entire range of LC-retarder voltages as shown in (Fig. 3(b)). To establish the relationship between the average intensity on the CCD camera image (b), which is proportional to the measured transmittance *T*_*exp*_ = *I*_*Air*_/*I*_0_, and to calculate the retardance using Eqn. 1, the intensity, *I*_*Air*_, at the “Air” ROI (out of sample) was measured. The incident light intensity *I*_0_ was controlled by electrical current in order to prevent saturation over a single LC-retarder cycle. The minimum intensity corresponded to the 0-wavelengths (or 1) while the maximum corresponded to 0.5-wavelengths (Fig. 4(a)). Since ${sin}^{2}\mathrm{2(}\theta -{\theta }_{R}\mathrm{)=1}$ by the selection of LC-retarder orientation (*θ*_*R*_ = ±*π*/4), the reference retardance of the LC-cell can be determined by ${T}_{exp}={sin}^{2}(\pi \Delta {n}_{LC}{d}_{LC}/\lambda )$ (Eqn. 1, where *n*_*LC*_, *d*_*LC*_ are the birefringence and thickness of the LC-cell, respectively (Fig. 3(c)).

The measured intensity was averaged over the ROI 2 × 2 pixels (see Fig. 4 inset). Regions on the LC-retarder and silk fibre are plotted in Fig. 4(a,b), respectively. It was necessary to select smaller ROIs due to a non-uniform thickness of the fibre, as described earlier, and to test the smallest integration area; potentially the noisiest signal. Since the “Air” region outside the silk fibre was used for reference retardance measurements, the transmittance follows Eqn. 1: ${T}_{\theta }={sin}^{2}(\pi \Delta nd/\lambda )$. Importantly, for the retardance corresponding to the half-wavelength Δ*nd*/*λ* = 0.5 the transmittance has a maximum (see arrow in Fig. 3(b)) and for the full wavelength Δ*nd*/*λ* = 1 it has a minimum (see arrow in Fig. 3(b)). This was expected and shows the validity of the employed calibration method. It was repeated for the different set of bandpass filters defining different wavelengths.

Figure 4(b) shows experimentally measured transmittance integrated over the 2 × 2 pixels ROI area on the silk fibre (rectangular box in the inset in (a)) *vs*. retardance of the LC-cell using the same procedure as used for the calibration of LC-retarder shown in (a). Even for a small number of the averaged pixels 2 × 2, a high confidence fit by $fit(x)=a{sin}^{2}(\pi [x\pm b])+o$ was obtained with *a*,*o* defining the amplitude and offset, *x* = Δ*n*_*LC*_*d*_*LC*_/*λ* is the retardance of LC-cell, and *b* = Δ*nd*/*λ* ± *m* where *m* = 0, 1, 2..., is determined by silk with the sign conventions: “+” for the LC-cell orientation as shown in the inset of (a) and “−” for the one perpendicular to that. The phase of the sin-wave was solely determined by the cumulative retardance through the LC-cell (“air”) and silk fibre Δ*nd*. However, when the retardance of the sample is exceeding one wavelength, it has an uncertainty of ±*m*. The best fit for the silk orientation shown in Fig. 4(b) was obtained with the “+” sign *fit*(*x*) = 110sin^2^(*π*(*x* + 0.35)) + 55 and corresponds to Δ*nd*/*λ* ± *m* = 0.35. The shaded region in Fig. 4(b) shows the ±5% change in Δ*n* around the best fit value. This qualitatively shows that a birefringence with ±2% difference can be distinguished. It is important to note that thickness and a change in birefringence equally affect the measured retardance. In this study, the thickness and orientation of the slow axis of the fibre did not affect the vertical offset of the sinusoidal curve in Fig. 4(b). The variation in *d* is the variation in retardance, therefore it changes the horizontal shift of the curve. Since the orientation of the slow axis is the first sinusoidal part of the Eqn. 1, it affects only the amplitude of the curve in Fig. 4(b). One possible explanation for the vertical offset is the depolarisation of the light due to the scattering at the surface of the silk fibre sample.

**Figure 5:**
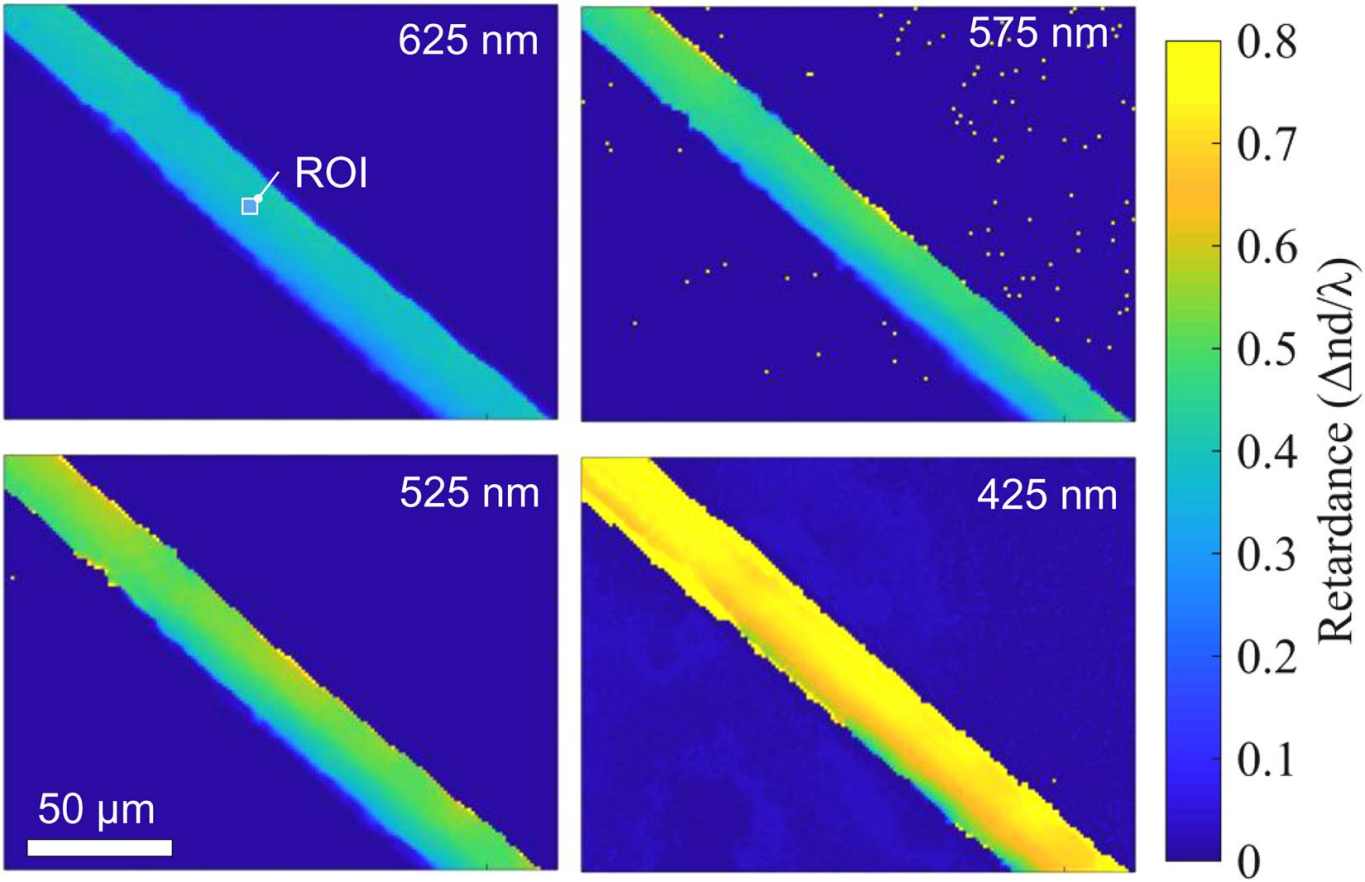
Retardance Δ*nd*/*λ* maps at four different wavelengths selected by interference filters (625, 575, 525, 425 nm) measured through the brown silk (*Antheraea pernyi*) single strand. Because silk naturally occurs as two interwoven strands, after degumming an asymmetry of the strand is revealed where the cross section has a trapezoidal or triangular shape. An average of 2 × 2 pixels was used for numerical processing of the original VGA 480 × 640 pixels CCD camera images. The optical resolution can be estimated by the radius of the Airy disk *w* = 0.61*λ*/*NA* = 0.95 *μ*m for the used *NA* = 0.4 objective lens at a wavelength of 625 nm. The silk fibre was placed parallel to the slow axis of the LC-retarder. The ROI was used to determine birefringence.

**Figure 6:**
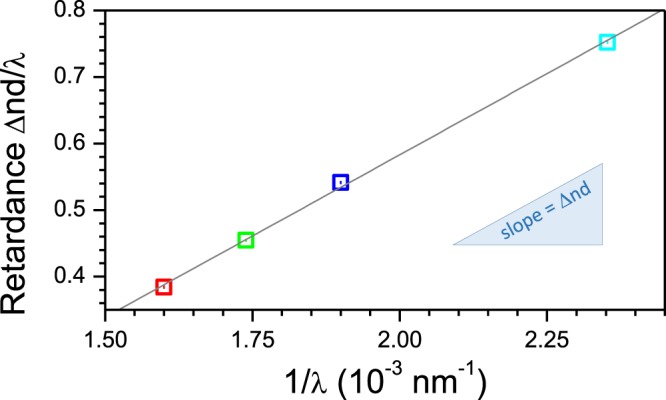
Retardance Δ*nd*/*λ vs*. 1/*λ* at four wavelengths (Fig. 5) 625, 575, 525, 425 nm presented by corresponding colour markers. Each point is an average over the ROI (see, Fig. 5). The linear fit equation *y* = *p*_1_*x* + *p*_2_, coefficient and 95% confidence interval are *p*_1_ = 487.5 (from 450.6 to 524.5), *p*_2_ = −0.3929 (from −0.4639 to −0.3219), *R*^2^ = 0.9994.

Retardance averaged over 2 × 2 pixels was determined for the entire image using the fitting method shown in Fig. 4(b). It is presented in Fig. 5 for the four different wavelengths selected by interference filters with 10 nm bandwidth. To obtain the exact Δ*n* value, retardance was measured at four wavelengths and Δ*nd*/*λ vs*. 1/*λ* was plotted, as shown in Fig. 6. A good linear fit was obtained for the retardance averaged over the ROI (Fig. 5) plotted in Fig. 6. Since the uncertainty of ±*m* affects only the vertical shift on the Δ*nd*/*λ vs*. 1/*λ* plot, the exact Δ*n* value can therefore be obtained. For the central part of the fibre, the birefringence Δ*n* ≈ (1.63 ± 0.05) × 10^−2^ was determined when *d* ≈ 30 *μ*m (for simplicity the silk fibre was assumed to be a cylinder).

**Figure 7:**
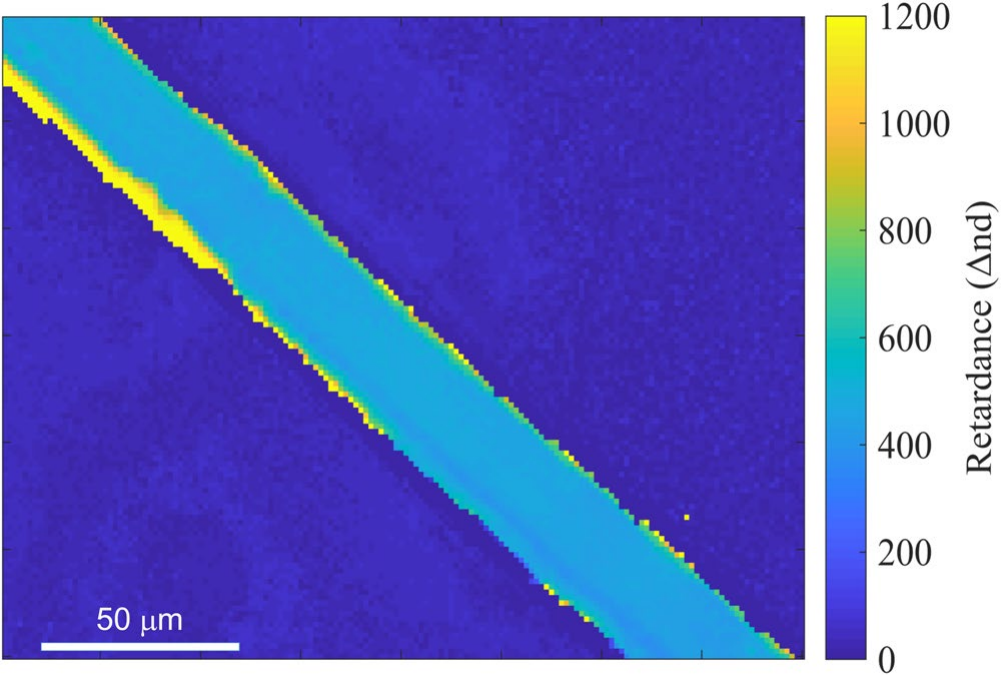
Wavelength-averaged retardance (Δ*nd* [nm]) map (averaged over the four wavelengths; Fig. 5). The thickness of the brown silk *Antheraea pernyi* fibre was *d* ≈ 30 *μ*m, which defines the average birefringence Δ*n* = (1.63 ± 0.05) × 10^−2^ at the centre of the silk fibre.

To obtain the map of the averaged retardance Δ*nd* [nm] over the spectral range from 425 nm to 625 nm, the same procedure as for Fig. 6 was carried out for each 2 × 2 pixels of the image at four wavelengths. From the slope of the linear fit, Δ*n* was calculated (as in Fig. 6) and is plotted in Fig. 7. Edges of the silk fibre scattered light stronger which resulted in a higher detected light intensity *T*_*θ*_ (Eqn. 1) and a corresponding two-fold increase in effective retardance.

## Conclusions and Outlook

In summary, the addition of a simple LC-retarder to a common polarisation microscopy set-up provides a highly sensitive method to image birefringence, as demonstrated for silk fibres. The proposed method relies on a large data set (sampling) of images obtained at different LC-retarder voltages (phase delays) used for the best fit. Here, it was shown that the birefringence of silk Δ*n* ≈ 1.6 × 10^−2^ could be determined with an uncertainty of just ∼±2% measured from an area of merely 2 × 2 pixels. Integration over larger ROI areas can be flexibly applied to achieve a better spatial resolution or an average birefringence, respectively. Measurements at several wavelengths were made to establish the absolute phase retardance. This is one advantage of our method over the commercial microscopy-based techniques for measuring birefringence which are carried out at merely one wavelength. The multi-wavelength measurement allows for extension of the retardance range beyond just one wavelength.

The technique for measuring birefringence established in this work, is simple and requires a fractional budget of ∼$2 k compared to the commerically established birefringence measurement tools. When the slow axis of the sample is unknown, or it is changing orientation over the image area, the axial alignment can be made by an additional measurement at four points (the minimum number required for the fit) of the angular orientation of the sample. Additionally, absorption anisotropy (diattenuation) can be measured using transmission with adequately high resolution ∼*λ* using this simple technique for analysis of molecular alignment^23^.

The proposed technique could also find application in the bio-medical field for cell monitoring and optical detection of cell division exploiting a new dimension, the birefringence, in addition to the usual set of the big-data dimensions of the lateral *xy*-position of the cell, time, intensity and shape of the object.
